# Radiography

**Published:** 1910-12-17

**Authors:** 


### SPECIAL ARTICLE.

### RADIOGRAPHY.

### X-RAYS IN THE DIAGNOSIS OF DISEASE OF THE LUNGS.

There is a great tendency nowadays to lay too much stress upon the efficacy of particular things—*x*-rays in diagnosis, vaccines in treatment, and so forth—to the exclusion of equally useful methods of diagnosis and measures of treatment. The new should be used as additions to, and not as replacements of, the old. Some very useful observations to this effect were made by Dr. Charles L. Minor in a paper read at a meeting of the Lænnec Society of the Johns Hopkins' Hospital, on February 28, 1910. Notwithstanding the opinions of many radiographers to the contrary, his own work has convinced him that really *early* phthisis gives abnormal physical signs before there are any abnormal shadows to the *x*-rays; that many cases of supposed *early* phthisis in which definite *x*-ray shadows are obtained are really examples of unsuspected and healed phthisis beginning to light up again; that scars from perfectly healed phthisis cast shadows indistinguishable from similar shadows due to caseous and active foci; that the *x*-rays are a most valuable aid in diagnosing lung cases; but that those who lay greater stress upon the *x*-rays than upon the other physical methods are liable to make serious mistakes.

He even goes so far as to say that as a means of early diagnosis the fluoroscope, while useful, is, save in the case of bronchial gland shadows—and it is noteworthy that phthisis does not as a rule give rise to caseous bronchial gland—by no means equal to an expert use of our standard physical diagnostic methods. He realises that many *x*-ray specialists will differ from this view, some maintaining that it is a far earlier means of diagnosis, but he is satisfied that this is chiefly due to the fact that the radiologist is rarely an expert physical diagnostician. In Germany, where several distinguished diagnosticians have used the *x*-rays freely, extreme claims for the early diagnostic value of the rays are not as common as in this country. Everyone is familiar with the ease with which the physician, unless he is specially interested in physical diagnosis, can overlook slight but significant changes observable by inspection, percussion, or auscultation. Hence the statement that any given case was negative on physical examination and showed shadows with the *x*-rays is of no great value, unless one knows who made the physical examination. One skilled radiologist, with no special physical diagnostic skill, made great claims of early diagnosis until he began to examine cases sent him by a notably careful physical diagnostician, when he was surprised to find that the superiority of his method over standard methods was lost.

Dr. Minor is quite candid and agrees that he has not found that the rays can antedate rough inspiration, feeble breathing, or slight vesiculo-bronchial breathing, as they must if they are to precede abnormal physical signs, and when it is recalled what are the pathological conditions which produce these slight changes it seems most improbable that they should cast shadows. If one asks, however, not whether they are a *very early* means of diagnosis, but whether they are a *valuable* means of diagnosis, then the answer must be most distinctly " yes "; they could not be given up without greatly lessening the information obtainable about any given case. Systematically used, they will be found to throw wonderful light upon lesions of the lungs, and there are few cases save the extremely incipient ones in which they are not useful.

#### Special Points in Technique.

There is no diagnostic measure in which it is more essential to be fully master of the use of the apparatus, and several months are necessary for the acquirement of this degree of use. Certain particular points may be given special mention.

First, one must be sure that the tube used has a good sharp focus, or else the result from it will be blurred and indistinct.

Second, it is absolutely necessary to have a convenient means of instantaneously raising or lowering the penetration of the tube without one's eye being taken from the screen. Otherwise, if the tube is of high vacuum, many fine details will be flooded out, just as with the microscope when too large a diaphragm is used, while if the vacuum be too low it will be impossible to penetrate the chest sufficiently to see anything well. It is, therefore, important always to use a tube of rather low vacuum with a multiple spark-gap or other proper apparatus inserted in the positive side of the circuit; in this way the light can be raised or lowered at will.

Third, there must be a proper diaphragm so as to cut down or enlarge the illuminated area, so that, after having first examined the picture of the whole thorax, a smaller portion can be isolated and studied alone, thus bringing out details otherwise invisible; this is especially important in examining and comparing the two apices.

Fourth, the fluorescent screen must be large enough to contain the shadow of the whole thorax at once; it should be at least 12 by 16 inches, and better, 16 by 24 inches. The cameras usually sold with the screens on the market are not good.

Fifth, for the safety of the operator, and to keep the room dark, the tube must be in a protective and opaque box, lined with lead, with an opening that can be diaphragmed down on one side, opposite the anode; and the tube must be held on a perfectly steady stand, which can be raised or lowered quickly and easily during use if the patient is to be examined erect, which is the position to be preferred.

Sixth, the patient should be at least two feet from the anode, and further is better as it lessens distortion. The transverse diameter of the patient's chest and the surface of the fluoroscope must be exactly at right angles to the course of the $x$-rays to avoid distortion, save during radiation made in an oblique position knowingly and on purpose.

Seventh, the doctor should spend at least five minutes in the absolutely darkened room before starting the machine so as to render his eye sensitive; just as in the case of a man going down a coal pit he is entirely unable to see properly at first, but gradually acquires his "pit eyes."

Eighth, a complete familiarity with the normal picture is essential if pathological changes are to be recognised and understood. A routine procedure should be followed in carrying out the examination, and the following sequence will be found useful: observe (1) the comparative size and shape of the two lung areas; (2) the course and angle of the ribs; (3) the sternal or mediastinal shadows (4) the position, size, and shape of the heart; (5) the movement of the bases; (6) the costo-diaphragmatic angle; (7) the size and clearness of the apices; (8) shadows in the body of the lung.

In studying the changes found in the lung in pulmonary tuberculosis it is wise to divide them into those found in incipient cases, those in moderately advanced cases, and those in very advanced cases. As regards really incipient cases, there may be no visible shadowing, although definite but slight abnormal physical signs are present. As Holzknecht well says, "The cases of apical tuberculosis, diagnosticated by the $x$-ray, are anatomically not cases of incipient tuberculosis, but old shrunken foci, only showing activity in spots. Such cases are clinically, but not anatomically, incipient tuberculosis. . . . The really anatomically incipient cases, with early tubercles in groups and catarrh, are radioscopically undeterminable."

The converse, that after a careful and painstaking examination of the lungs no abnormal physical signs are to be found in some cases that show distinct shadows, merely indicates that the healing of phthisis does not render the affected areas transparent to the rays again.

Usually, however, the careful examiner will find shadows and changes on auscultation and percussion co-existing and corroborating each other, and it is this corroboration which the $x$-ray brings to the results of our regular examination that is one of its most valuable features.

